# Anti-synthetase Syndrome Presenting Primarily as Interstitial Lung Disease in a Young Adult: A Diagnostic Challenge in Acute Medicine

**DOI:** 10.7759/cureus.97344

**Published:** 2025-11-20

**Authors:** Kawser Ahmed, Faaraan Bangash, Arsalan Bangash, Yaseen Hussain

**Affiliations:** 1 Acute Internal Medicine, Northampton General Hospital NHS Trust, Northampton, GBR; 2 Acute Medicine, Northampton General Hospital NHS Trust, Northampton, GBR; 3 General Medicine, Northampton General Hospital NHS Trust, Northampton, GBR

**Keywords:** anti-synthetase (as) syndrome, auto immune, diffuse interstitial lung disease, home oxygen therapy, lung transplant

## Abstract

Anti-synthetase syndrome (ASyS) is a rare autoimmune connective tissue disorder (CTD) characterized by the presence of autoantibodies targeting tRNA synthetase, most notably the anti-Jo1 antibody. Clinically, it typically manifests with interstitial lung disease (ILD), inflammatory myopathy, arthritis, Raynaud’s phenomenon, and the so-called mechanic’s hands. Diagnosis can be challenging due to clinical overlap with other pulmonary and rheumatological disorders.

We report a case of a young woman in her early 20s who presented at our same day emergency care (SDEC) with a four-week history of exertional dyspnea, low-grade fever, and productive cough, initially managed as a lower respiratory tract infection in the community. Persistent symptoms prompted further evaluation, and CT imaging of the chest, abdomen, and pelvis revealed bilateral ground-glass opacities suggestive of ILD. Five days later, she re-presented with hemoptysis, worsening breathlessness, new-onset myalgia, arthralgia, facial erythema, and hyperkeratosis of the fingertips. Autoimmune screening revealed strongly positive anti-Jo-1 and anti-Ro52 antibodies, confirming the diagnosis of anti-synthetase syndrome. She was treated with intravenous (IV) pulse methylprednisolone, mycophenolate mofetil, and rituximab, with partial clinical improvement and ongoing follow-up for advanced ILD management in a tertiary center. This case highlights how an initial diagnosis of lower respiratory tract infection (LRTI) may mask an underlying autoimmune syndrome (anti-synthetase syndrome), which later manifests as progressive respiratory failure requiring lung transplant.

Therefore, as acute medicine physicians, it is imperative to maintain a broad differential diagnosis, particularly in young females newly diagnosed with possible ILD. There may be underlying conditions that require careful examination by acute care physicians, especially in the presence of systemic symptoms, notably rheumatological symptoms. This vigilance is crucial to avoid delayed diagnosis of anti-synthetase syndrome, which necessitates early multidisciplinary involvement to achieve better outcomes.

## Introduction

Anti-synthetase syndrome (ASyS) is a rare autoimmune disorder characterized by the presence of autoantibodies targeting aminoacyl-tRNA synthetases (anti-ARS), including anti-Jo1, anti-PL7, anti-PL12, anti-EJ, anti-OJ, anti-KS, anti-O, and anti-YRS/HA. This condition presents with a diverse range of symptoms affecting various organs, most commonly the muscles, lungs, joints, and skin [1]. Women are more likely to be affected than men, and the estimated ratio is about 2:1. The prevalence in the UK is approximately 1.5 cases per 100,000 population, though true figures are likely underestimated due to diagnostic variability [2].

The syndrome was first described by Marguerie et al. in 1990, who identified a distinct subgroup of patients with anti-Jo-1 antibodies and a clinical triad of myositis, interstitial lung disease (ILD), and arthritis. Since then, several antisynthetase autoantibodies and clinical variants have been recognized, contributing to diagnostic complexity and potential delays in recognition [3]. Among its manifestations, interstitial lung disease is the most common and serious, often determining prognosis and occasionally presenting as the sole clinical feature [4]. Early diagnosis is therefore critical, as timely initiation of corticosteroids and immunosuppressive agents - such as azathioprine, mycophenolate mofetil, or rituximab - can improve long-term outcomes [5].

We report the case of a young adult who presented with isolated respiratory symptoms and radiological findings suggestive of interstitial lung disease, later diagnosed as anti-synthetase syndrome following the development of systemic features. This case highlights the diagnostic challenges associated with atypical presentations and underscores the importance of early multidisciplinary collaboration and autoimmune screening in unexplained interstitial lung disease.

## Case presentation

A young woman in her early 20s presented to the Same Day Emergency Care (SDEC) unit with a four-week history of exertional dyspnea, low-grade fever, and a productive cough with yellow sputum. Upon systemic inquiry, she denied experiencing hemoptysis, chest pain, palpitations, calf pain, rash, or leg swelling. She also reported no symptoms related to the abdominal, urinary, or musculoskeletal systems. Her personal history revealed no prior medical conditions; she was a non-smoker, did not consume alcohol, and had never used recreational drugs. She lives with the family and works at KFC. She had recently returned from travel to her home country in West Africa. Initially, she was treated in the community with oral amoxicillin for a presumed lower respiratory tract infection, but this did not result in improvement. Of note she had no family history of lung cancer or thromboembolic diseases.

**Figure 1:**
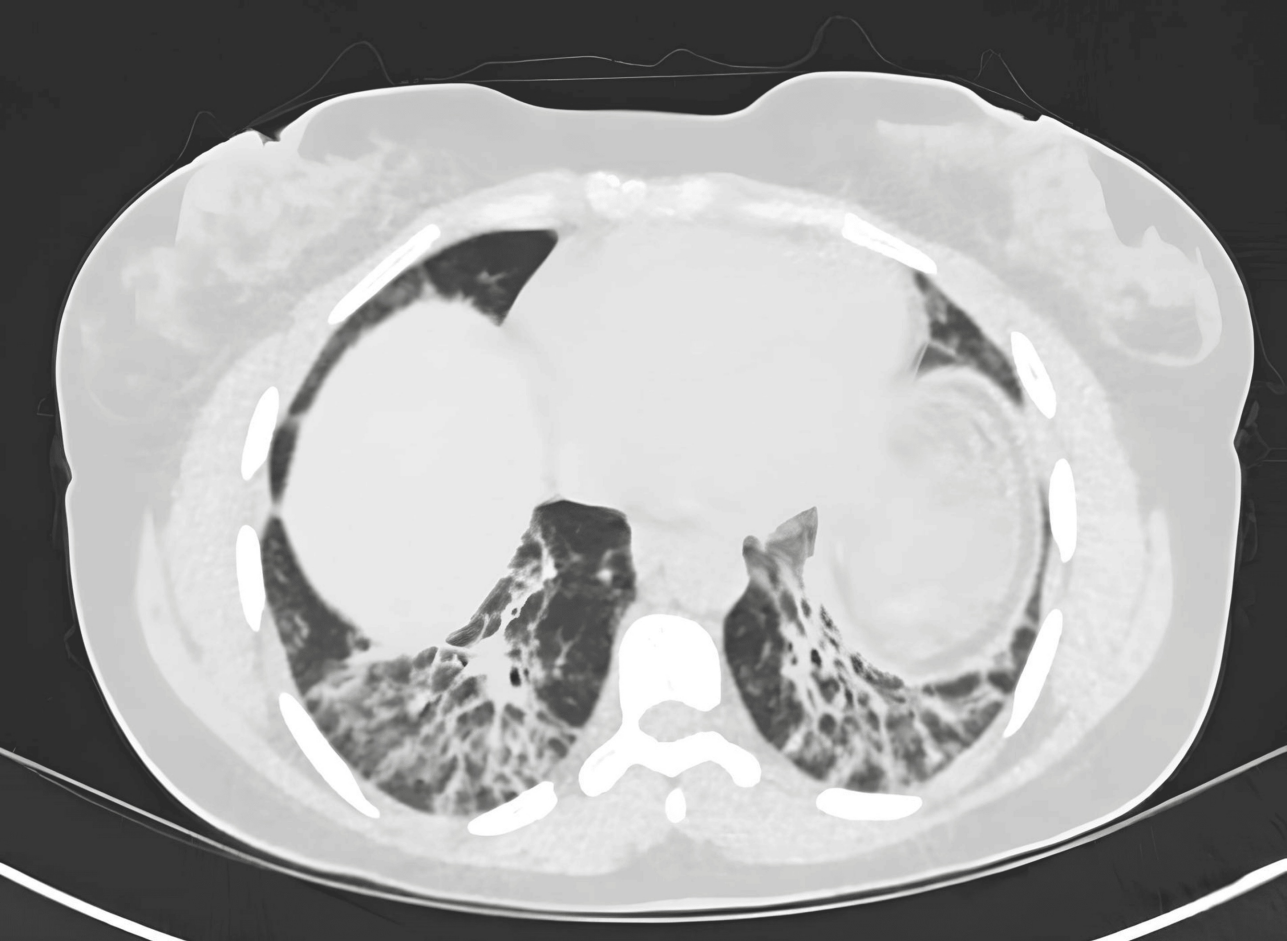
CT Thorax is showing bilateral patchy consolidation with ground-glass opacities, suggesting differential diagnoses of organizing pneumonia, chronic eosinophilic pneumonia, COVID-19, or atypical pneumonia. CT = Computed Tomography

Computed tomography (CT) thorax with contrast demonstrated extensive patchy areas of consolidation and ground-glass opacification involving both lungs, most pronounced in the lower lobes. The abnormalities exhibited a predominantly peripheral and subpleural distribution, with a few perihilar regions also affected. The tracheobronchial tree appeared patent, and there was no evidence of pleural effusion. Additionally no significant mediastinal and hilar lymphadenopathy was identified. There was no radiological evidence of a large central pulmonary embolus, and no aggressive osseous lesions were observed (Figure 1).

Referrals were made to respiratory and rheumatology specialists, who recommended conducting autoimmune screenings, including antinuclear antibody (ANA), extractable nuclear antigen (ENA) panel, antineutrophil cytoplasmic antibodies (ANCA), anti-double-stranded DNA (anti-dsDNA), tuberculosis (TB) screening, COVID-19, and atypical pathogen screening. Appointments were scheduled in each respective clinic. As the patient was hemodynamically stable, she was discharged with plans for outpatient follow-up pending results from both the respiratory and rheumatology teams.

Five days following her initial presentation, the patient experienced worsening dyspnea and newly onset hemoptysis, accompanied by myalgia in the upper limbs, proximal muscle weakness, and arthralgia affecting the shoulders, elbows, and knees. Her National Early Warning Score (NEWS) was recorded at 7, with an oxygen saturation of 88% on room air, a heart rate of 109 bpm, and a temperature of 38.2 °C. Arterial blood gas analysis indicated type 1 respiratory failure (Table 1).

**Table 1:** Arterial blood gas (ABG) and electrolyte analysis on room air (FiO₂ 21%) showing mild metabolic acidosis (pH 7.33, base excess -7.5, bicarbonate 18.9 mmol/L) with hypoxemia (pO₂ 7.5 kPa, sO₂ 88.4%), consistent with type I respiratory failure.

Parameter	Result	Unit	Normal Reference Range
pH	7.33	—	7.35 – 7.45
pCO₂	4.53	kPa	4.7 – 6.0
pO₂	7.5	kPa	10 – 13 (on air)
sO₂	88	%	94 – 98
O₂Hb	87.1	%	94 – 98
COHb	0.9	%	<1.5 (non-smoker), <5 (smoker)
MetHb	0.6	%	<1.5
FiO₂	21.0	%	—
tHb	145	g/L	120 – 160 (female) / 130 – 170 (male)
Base Excess (BE)	-7.5	mmol/L	-2 to +2
Standard Bicarbonate (SBC)	18.9	mmol/L	22 – 26
Lactate	1.3	mmol/L	0.5 – 2.0
Total Oxygen Content (tO₂)	7.9	mmol/L	8.0 – 11.0
p50	3.62	kPa	3.5 – 3.6
Temperature	37.0	°C	—
Sodium (Na⁺)	134	mmol/L	135 – 145
Potassium (K⁺)	3.5	mmol/L	3.5 – 5.0
Ionized Calcium (Ca²⁺)	1.16	mmol/L	1.12 – 1.32
Chloride (Cl⁻)	109	mmol/L	98 – 106
Glucose	4.4	mmol/L	3.5 – 7.8

A general examination revealed hyperkeratotic and cracked skin on the fingertips, along with erythema of the cheeks. Chest auscultation findings remained unchanged. Blood tests showed a creatine kinase (CK) level of 832 U/L and an elevated lactate dehydrogenase (LDH) level of 505 U/L, with normal liver enzymes and stable inflammatory markers. Based on her Wells score of 7, a CT pulmonary angiogram was performed, which excluded pulmonary embolism and indicated no interval change from previous imaging. However, due to her oxygen requirement and high NEWS, she was admitted under the acute internal medicine team. Meanwhile, her autoimmune screening returned positive for anti-Jo1 and anti-Ro52 antibodies (Table 2).

**Table 2:** Laboratory results of second presentation demonstrate raised inflammatory markers (CRP, ESR, LDH), elevated muscle enzymes (CK), mild transaminitis (ALT), hyponatremia, and low calcium levels. Cardiac markers (troponin) were elevated. COVID-19 PCR = coronavirus disease-19 polymerase chain reaction; atypical screening = urinary legionella antigen and mycoplasma serology.

Parameter	Result	Reference Range
Hemoglobin (Hb)	135 g/L	120–150 g/L
Hematocrit (Hct)	41%	36–46%
White Cell Count (WCC)	4.6 × 10⁹/L	4–10 × 10⁹/L
Neutrophil Count	2.10 × 10⁹/L	1.8–7.4 × 10⁹/L
Platelet Count	233 × 10⁹/L	150–400 × 10⁹/L
C-Reactive Protein (CRP)	56 mg/L ↑	<5 mg/L
Sodium (Na⁺)	130 mmol/L ↓	133–146 mmol/L
Potassium (K⁺)	4.5 mmol/L	3.5–5.3 mmol/L
Urea	4.4 mmol/L	2.5–7.8 mmol/L
Creatinine	41 μmol/L ↓	45–84 μmol/L
Estimated Glomerular Filtration Rate (eGFR)	>90 mL/min	—
Total Protein	68 g/L	60–80 g/L
Albumin	36 g/L	35–50 g/L
Corrected Calcium (cCa²⁺)	2.19 mmol/L ↓	2.25–2.60 mmol/L
Total Bilirubin	7 μmol/L	0–21 μmol/L
Alkaline Phosphatase (ALP)	37 IU/L	30–130 IU/L
Alanine Aminotransferase (ALT)	50 IU/L ↑	5–33 IU/L
International Normalized Ratio (INR)	1.0	0.8–1.2
Activated Partial Thromboplastin Time (APTT)	30 sec	22–30 sec
D-Dimer	760 ng/mL ↑	<500 ng/mL
NT-proBNP	58 ng/L	0–400 ng/L
Creatine Kinase (CK)	832 IU/L ↑	25–200 IU/L
Lactate Dehydrogenase (LDH)	505 IU/L ↑	135–214 IU/L
Erythrocyte Sedimentation Rate (ESR)	24 mm/h ↑	1–12 mm/h
Vitamin D	<20 nmol/L (Deficient)	≥30 nmol/L (Sufficient)
Troponin	58 ng/L ↑	<15 ng/L
Quantiferon	Negative	-
COVID-19 PCR	Negative	-
Atypical screening	Negative	-

**Figure 2:**
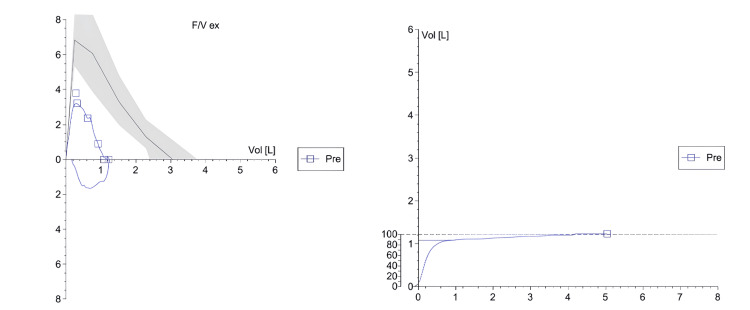
Spirometry demonstrating a restrictive ventilatory defect. Spirometry reveals a reduced forced vital capacity (FVC) and forced expiratory volume in the first second (FEV₁) with preserved FEV₁/FVC ratio (~88%), consistent with a restrictive pattern. The reduced total lung volumes and low % predicted values suggest significant impairment in lung expansion, aligning with the patient’s known interstitial lung involvement.

Consequently, the acute medicine team maintained continuous communication with the rheumatology and respiratory teams as well as cardiology team for mild elevation of troponin. Rheumatology recommended an MRI of the upper limbs, as well as nerve conduction studies (NCS) and electromyography (EMG) of the upper limb. Her nerve conduction study was normal, and the needle EMG did not demonstrate any features of myopathy or myositis. Interventional radiology performed a deltoid muscle biopsy as advised by rheumatology. Histology showed non-inflammatory infiltrates, which rheumatology attributed to early steroid treatment. The respiratory team conducted pulmonary function testing, which demonstrated severe airflow limitation and significantly reduced alveolar volume, with preserved gas transfer efficiency (normal KCO), consistent with an ILD pattern (Figure 2).

The respiratory team placed her on a two-week pathway in the ILD meeting under the respiratory clinic. Cardiology team performed an inpatient echocardiography which showed bright pericardium and trace pericardial effusion at the left ventricular inferolateral wall; otherwise remainder of study was normal. Cardiologist gave their opinion as type 2 myocardial infarction due to hypoxia and recommended to manage the primary etiology. 

Based on the patient's clinical presentation, positive autoimmunity, and severe pulmonary fibrosis, the multidisciplinary team (MDT) concluded a diagnosis of anti-synthetase syndrome. The consensus was to initiate treatment with intravenous pulse methylprednisolone at a dosage of 1 g daily for three days, in conjunction with mycophenolate mofetil (MMF) and subsequently with rituximab (RTX), despite the patient being scheduled for MRI, NCS, and EMG at that time. Following three days of treatment, the patient exhibited no hemoptysis, but continued to experience a persistent cough, shortness of breath (SOB), and a requirement for supplemental oxygen. Consequently, the MDT commenced long-term steroid therapy (prednisolone) at 1 mg/kg (50 mg, given the patient's weight of 50 kg) for an additional two days, followed by 40 mg for two weeks. The dosage was then reduced by 10 mg every two weeks until reaching 20 mg once daily, followed by a reduction of 2.5 mg every two weeks until reaching 10 mg once daily, and subsequently reduced by 1 mg weekly. Concurrently, the patient received MMF 500 mg twice daily, hydroxychloroquine sulfate 200 mg daily, colecalciferol 50,000 units weekly for six weeks, followed by colecalciferol with calcium carbonate 1500 mg/400 units daily, and omeprazole 20 mg daily. The home oxygen team was engaged prior to discharge to evaluate the necessity for long-term home oxygen therapy. After thorough assessment, the patient was discharged with arrangements for long-term home oxygen. Follow-up was scheduled with the acute medicine virtual ward team, rheumatology, and respiratory team at one, three, and six months. The respiratory team included the patient in the ILD MDT, which considered a referral to the ILD clinic at Glenfield Hospital, UK, for lung transplantation. They also recommended increasing the MMF dose to 1 g twice daily, hydroxychloroquine 200 mg twice daily, and advised repeat CT and forced vital capacity (FVC) monitoring, with continued follow-up in their connective tissue disease (CTD) clinic. Glenfield Hospital placed the patient on the waiting list for early lung transplantation as she was not responding to the above regimens as expected. All team members were informed to ensure the patient remained up-to-date with vaccinations, pneumocystis carinii pneumonia (PCP) prophylaxis, and to refrain from discontinuing any disease-modifying antirheumatic drugs (DMARDs) without consulting the transplant team. At the time of writing this case report, the patient was alive and in reasonably good health, maintained on long-term home oxygen and the above-mentioned regular medication, with the specified follow-up schedules.

## Discussion

ASyS is a rare autoimmune disorder classified within the spectrum of idiopathic inflammatory myopathies (IIMs) [6]. It is characterized by the presence of antibodies against aminoacyl tRNA synthetase, most commonly the anti-Jo1 antibody [7]. The disease exhibits significant clinical heterogeneity, typically manifesting as ILD, inflammatory myopathy, arthritis, Raynaud's phenomenon, and hyperkeratotic mechanic's hands [8].

A significant diagnostic challenge arises from the incomplete presentation of features, as 5% of patients only exhibit the full triad of ILD, myositis, and arthritis at onset [6]. This diagnostic uncertainty was evident in our case: the patient initially presented with respiratory symptoms that mimicked an infection. The absence of fever, minimal systemic inflammation, and lack of response to antibiotics were early, albeit subtle, indicators suggesting a non-infectious process. High-resolution computed tomography (HRCT) demonstrating bilateral ground-glass opacities further indicated a potential interstitial pathology (organizing pneumonia). 

From a diagnostic perspective, this case highlights the significance of systematic exclusion and evolving recognition. Once infectious and malignant causes of ILD were excluded, the subsequent emergence of myalgia, arthralgia, facial erythema, and fingertip hyperkeratosis suggested a systemic autoimmune process. The subsequent identification of anti-Jo-1 and anti-Ro-52 antibodies confirmed the diagnosis of ASyS. Notably, anti-Ro-52 co-positivity has been associated with enhanced activation of type 1 interferon pathways, which promote macrophage-driven alveolar injury and progressive pulmonary fibrosis, thereby mechanistically explaining the patient's severe, treatment-resistant ILD [9].

Pulmonary involvement is the most frequent and life-threatening manifestation of ASyS, occurring in up to 70-100% of patients [10]. Pathophysiologically, immune-mediated injury leads to alveolar epithelial damage, macrophage activation, and fibroblast proliferation, culminating in fibrotic remodeling. If unchecked, this cascade can result in irreversible architectural distortion, as observed in this patient who progressed to end-stage fibrotic lung disease despite aggressive immunosuppression [11]. In this patient, despite early initiation of intravenous pulse methylprednisolone and MMF, disease progression necessitated escalation to rituximab, an anti-CD20 monoclonal antibody that has shown efficacy in acute onset or exacerbation of ILD [12]. Unfortunately, she developed advanced pulmonary fibrosis requiring long-term oxygen therapy and subsequent referral to a tertiary transplant center for lung transplantation, underscoring the association between anti-Ro52 positive in ASyS with more severe form of ILD [13].

This case is particularly noteworthy for several reasons. First, it illustrates how ASyS can initially mimic a benign respiratory infection, thereby delaying recognition and definitive management. Second, it demonstrates the dynamic evolution of symptoms, where extrapulmonary features appeared later and became diagnostic clues, highlighting the need for repeated and holistic reassessment in undiagnosed ILD. Third, the coexistence of anti-Jo-1 and anti-Ro-52 antibodies, with severe progressive ILD despite multimodal immunosuppression, is significant. Fourth, the case emphasizes the need for early autoimmune screening in young, previously healthy patients with unexplained ILD, as early therapy may alter the natural course of the disease.

## Conclusions

ASyS represents a rare but potentially life-threatening cause of interstitial lung disease, particularly when diagnosis is delayed due to nonspecific respiratory symptoms. This case highlights the importance of maintaining diagnostic vigilance for autoimmune causes in young patients with unexplained interstitial lung disease. Early identification through serological testing and multidisciplinary collaboration is essential to initiate timely immunosuppressive therapy and prevent irreversible pulmonary fibrosis. Awareness of aggressive phenotypes, especially in anti-Ro52-positive patients, is crucial for prompt referral to specialist centers, including consideration for lung transplantation when appropriate.
